# Continuous or interrupted suture technique for hepaticojejunostomy? A national survey

**DOI:** 10.1186/s12893-018-0418-z

**Published:** 2018-10-11

**Authors:** Maximilian Brunner, Jessica Stockheim, Christian Krautz, Dimitrios Raptis, Stephan Kersting, Georg F. Weber, Robert Grützmann

**Affiliations:** Department of General Surgery, University Hospital of Friedrich-Alexander-University, Krankenhausstraße 12, 91054 Erlangen, Germany

**Keywords:** Hepaticojejunostomy, Pancreatic surgery, Hepatic surgery, Surgical technique, Survey

## Abstract

**Background:**

Hepaticojejunostomy is commonly used in hepato-bilio-pancreatic surgery and a crucial step in many surgical procedures, including pancreaticoduodenectomy. The most frequently used techniques are the interrupted suture and the continuous suture technique. Currently, there is no data available in regard to the utilization of these techniques.

**Methods:**

In total, 102 hospitals in Germany were invited between September and November 2017 to participate in this survey. Using a paper-based questionnaire, data were collected on surgical technique and complication rates of hepaticojejunostomies.

**Results:**

A total of 77 of the 102 addressed hospitals (76%) participated in the survey. On average, each hospital performed 71 hepaticojejunostomies per year - most often in the context of pancreaticoduodenectomy (71%). 24 (31%) hospitals exclusively use an interrupted suture technique, 7 (9%) hospitals solely a continuous suture technique, 3 (4%) hospitals perform a combination of continuous and interrupted suture technique and 43 (56%) hospitals decide on one of both techniques depending on intraoperative findings. According to the participants in this survey, the continuous suture technique is significantly faster than the interrupted suture technique in hepaticojejunostomy (*p* = 0,015). There were no significant differences in the overall complication rate (*p* = 0,902) and insufficiency rate (*p* = 1,000).

**Conclusions:**

In Germany, there is a heterogeneity in the technique used to create a hepaticojejunostomy. As our survey suggests that the use of continuous suture technique may offer an advantage in time without jeopardizing patient outcomes, the different techniques should be compared in a randomized controlled study.

## Background

The surgical technique of the hepaticojejunostomy represents the “surgical school” in a unique way and while some of us use either continuous or interrupted sutures depending on the situation and the operative situs, others adhere very much to their surgical education be it interrupted or continuous suturing for all cases.

Hepaticojejunostomies represent an important step in pancreatic resections, liver resections, liver transplantations and bile duct resections, are used as a palliative procedure for non-resectable tumors of the pancreatic head and distal bile duct and are performed in bile duct injuries. Failure of this anastomosis leads to considerable morbidity and even mortality [1, 2].

After various methods of anastomosing the biliary system with the gastrointestinal tract (cholecystocolostomy, cholecystojejunostomy, hepaticoduodenostomy) had been published at the end of the nineteenth century, Dahl was the first to report a hepaticojejunostomy in 1909 [3–6]. Over the years, various modifications have been described [7]. Since then, hepaticojejunostomy has been established as an important component of many surgical procedures and all other techniques have been more or less abandoned.

Basic principles for the successful implementation of a hepaticojejunostomy are [8]:

- A tension-free reconstruction
- Anastomosis in the area of ​​intact, well-perfused bile duct and small bowel mucosa
- Precise mucosal adaptation between the bile duct and jejunum
- Creation of hepaticojejunostomy near to the hepatic duct bifurcation

The most important complications following a hepaticojejunostomy are bile duct leakage and anastomotic stenosis. In the literature leakage rates after hepaticojejunostomies vary between 2.3 and 5.6% [9, 10]. Although this is a relatively rare postoperative complication, bile duct leakage can have far-reaching consequences with a high risk of prolonged hospitalization and need for interventional drainage or re-laparotomy, which is associated with high morbidity and mortality, even in high volume centers [1, 2]. For the development of anastomotic stenosis, studies report rates between 3.7 and 8.0% [11, 12].

**Fig. 1:**
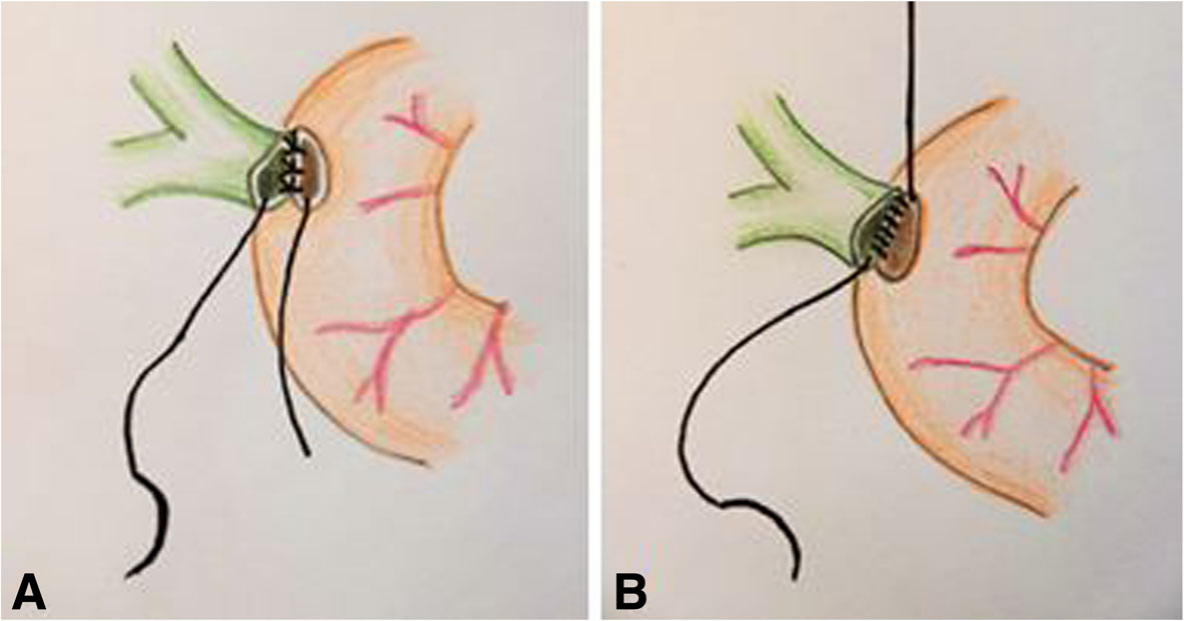
Hepaticojejunostomy with interrupted suture technique (**a**) and continuous suture technique (**b**); own figures

**Fig. 2:**
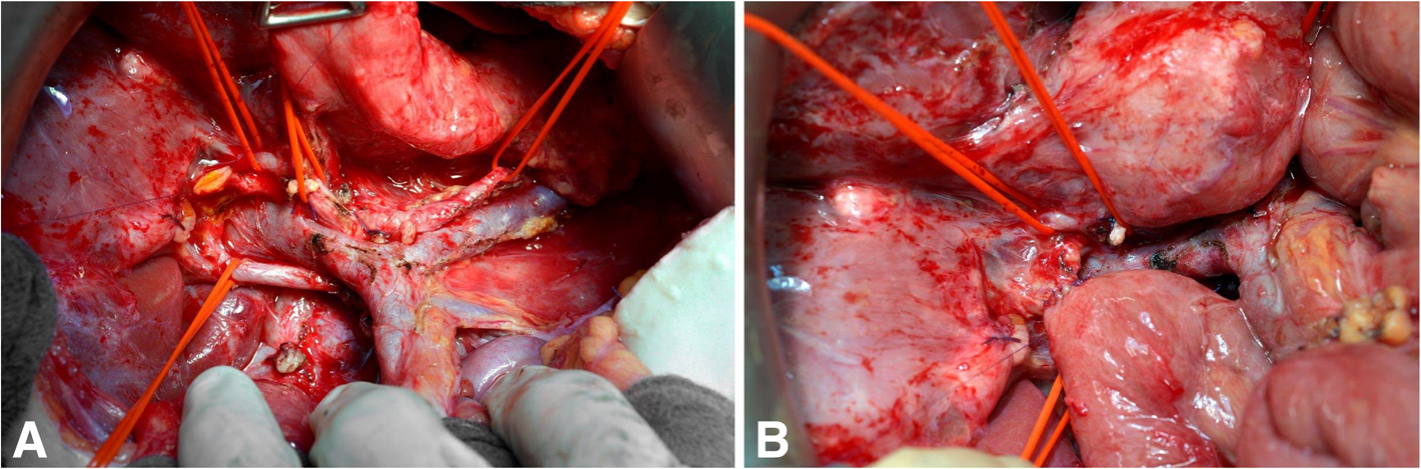
Hepaticojejunostomy with interrupted suture technique; intraoperative pictures: situs after pancreaticoduodenectomy and before hepaticojejunostomy (**a**) and situs after hepaticojejunostomy in interrupted suture technique (**b**); pictures are examples for the interrupted suture technique from our institute, other versions of the technique are possible

**Fig. 3:**
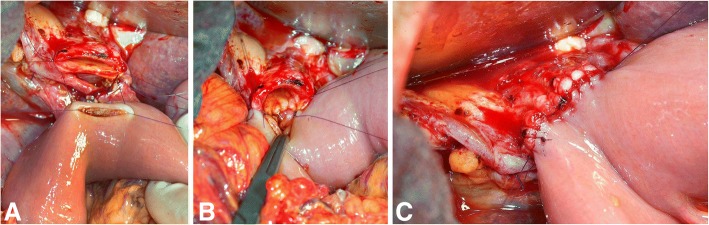
Hepaticojejunostomy with continuous suture technique; intraoperative pictures: situs before hepaticojejunostomy (**a**), situs after reconstruction of the posterior wall in continuous suture technique (**b**) and situs after complete hepaticojejunostomy in continuous suture technique (**c**); pictures are examples for the continuous suture technique from our institute, other versions of the technique are possible

There are various surgical techniques available for the creation of a hepaticojejunostomy. Figures 1, 2 and 3 show the most commonly used techniques: interrupted suture technique and continuous suture technique. A combination of both techniques is also possible (posterior and anterior wall in different techniques). The advantage of the interrupted suture technique is the universal use even for small bile ducts, whereas the costs and the operating time for this technique should be higher in comparison to the continuous suture technique (Table 1). Especially for larger bile ducts, the continuous technique might offer a better sealing of the anastomosis. Conversely, advocates of the interrupted technique allege that the continuous suture might lead in long term to a higher rate of stenosis at the anastomosis.

**Table 1:** Advantages and disadvantages of interrupted suture technique and continuous suture technique during hepaticojejunostomy

	Interrupted suture technique	Continuous suture technique
Advantages	Always possible	Lower costs
		Shorter operating time
Disadvantages	Higher costs	Difficult for very small bile ducts
	Longer operating time	

Despite the frequent necessity of hepaticojejunostomies in surgery and the relevant consequences for the patient with leakage or stenosis, there are no randomized studies to compare the different surgical techniques.

In preparation of a randomized trial, the aim of the current questionnaire-based survey was to determine the status quo of the surgical techniques used for hepaticojejunostomies in Germany.

## Methods

In September 2017, a total of 102 surgical hospitals in Germany were addressed to take part in this survey. Since most hepaticojejunostomies are constructed as part of pancreatic surgery and these are more likely to be performed in larger institutions, all hospitals in Germany that treat least 30,000 cases per year were selected for inclusion in this survey. In November 2017, a reminder letter was sent to all hospitals that had not responded by then. The collection of data was paper-based to make the answer to the questionnaire as simple as possible.

In the questionnaire the following aspects were queried:

- Number of hepaticojejunostomies per year
- Surgical technique used for hepaticojejunostomy
- Criteria for the choice of technique (if several techniques were used)
- Sutures used for hepaticojejunostomy
- Estimated duration of hepaticojejunostomy
- Estimated overall complication rate after hepaticojejunostomy
- Estimated leakage rate after hepaticojejunostomy

### Statistical analysis

The statistical analysis of the collected data was done using the SPSS statistical program package (SPSS inc., Chicago, USA). To compare categorical data, the chi-square test was used. For comparison of quantitative data the Mann-Whitney U-test or the t-test were used. A *p*-value of less than 0.05 was considered significant.

## Results

Of the 102 German surgical hospitals addressed, 77 hospitals (25 university hospitals (33%), 52 other hospitals (68%)) responded. The average number of hepaticojejunostomies performed per year was 71 [range 17–300]. Open surgical approach was used for all hepaticojejunostomies. Hepaticojejunostomies were performed with a significantly higher frequency in university hospitals than in other hospitals (115 vs. 51 on average, *p* <  0.001). Mostly hepaticojejunostomies were done during pancreatic resections (71%), followed by bile duct resections (15%) and liver resections (14%) (Table 2).

**Table 2:** Characteristics of the participating hospitals

Response rate		77 / 102 (76%)
Hospitals	University hospitals	25 / 76 (33%)
	Other hospitals	52 / 76 (68%)
Mean number of hepaticojejunostomies per year [range]	All	71 [17–300]
		median 54
	- University hospitals	115 [40–300]
	- Other hospitals	51 [17–190]
Hepaticojejunostomies during ... (in %) [range]	Pancreatic resection	71 [40–100]
	Bile duct resection	15 [0–40]
	Liver resection	14 [0–49]
	Other surgical procedures	1 [0–33]

Depending on the individual situation, most hospitals (56%) use both, either the interrupted suture technique or the continuous suture technique, to create a hepaticojejunostomy. 31% of the hospitals always apply an interrupted suture technique, whereas 9% always utilize a continuous suture technique. Only 4% use a combination of both techniques in the same anastomosis (Table 3). The surgical technique used for hepaticojejunostomy did not differ between university hospitals and other hospitals (*p* = 0.620) and between hospitals above and below the median of 54 hepaticojejunostomies per year (*p* = 0.833). Hospitals using both suturing techniques indicated in 95% of the cases the bile duct diameter, in 37% the bile duct wall thickness and in 26% other reasons to be criteria for the choice of technique. Other decision criteria were: surgeon’s preference, the presence of infection, the quality of exposure of the site, the extent of surgery, the location of the anastomosis (central vs. peripheral), the underlying diagnosis, the age of the patient (pediatric vs. adult) and whether it is a redo procedure.

**Table 3:** Techniques of hepaticojejunostomy; HJ = hepaticojejunostomies

		All (*n* = 76)	University hospitals (*n* = 24)	Other hospitals (*n* = 52)	*p*-value	Hospitals with < 54 HJ/year (*n* = 38)	Hospitals with ≥54 HJ/Jahr (*n* = 39)	*p*-value
Technique used	Interrupted suture technique	24 (31%)	10 (40%)	14 (27%)	0,620	11 (29%)	13 (33%)	0,833
	Continuous suture technique	7 (9%)	1 (4%)	6 (12%)		3 (8%)	4 (10%)	
	Interrupted + continuous suture technique	43 (56%)	13 (52%)	30 (58%)		23 (61%)	20 (51%)	
	Combination of interrupted and continuous suture technique	3 (4%)	1 (4%)	2 (4%)		1 (3%)	2 (5%)	
Technique used in cases of S + C (%) [range]	Interrupted suture technique	48 [5–95]	49 [10–90]	48 [5–95]	1,000	47 [5–95]	49 [5–90]	1,000
	Continuous suture technique	52 [5–95]	51 [10–90]	52 [5–95]		53 [5–95]	51 [10–95]	
Decision criteria for the choice of technique (in cases of I + C)*	Bile duct diameter	41 (95%)	12 (92%)	29 (97%)		22 (96%)	19 (95%)	
	Bile duct wall thickness	16 (37%)	6 (46%)	10 (33%)		8 (35%)	8 (40%)	
	Other reason	11 (26%)	6 (46%)	5 (17%)		3 (17%)	7 (35%)	
Suture material used*	Monofilament suture	76 (100%)	24 (100%)	52 (100%)	**< 0,001**	38 (100%)	39 (100%)	0,052
	Absorbable suture	76 (100%)	24 (100%)	52 (100%)		38 (100%)	39 (100%)	
	Strength 3.0	1 (1%)	0 (0%)	1 (2%)		1 (3%)	0 (0%)	
	Strength 4.0	26 (34%)	4 (16%)	22 (42%)		15 (39%)	11 (28%)	
	Strength 5.0	60 (78%)	23 (92%)	37 (71%)		25 (66%)	35 (90%)	
	Strength 6.0	17 (22%)	13 (52%)	4 (8%)		4 (8%)	14 (36%)	

*Multiple answers possible

Interestingly, all of the hospitals surveyed uniformly use monofilament absorbable sutures for the hepaticojejunostomy. University hospitals used significantly thinner sutures than other hospitals (*p* <  0.001) (Table 3).

The duration of the continuous suture technique was estimated to be significantly shorter than the time estimated for the interrupted suture technique (*p* = 0.002). Regarding the estimated overall complication rate and leakage rate, there were no significant differences between the techniques (*p* = 0.695 and *p* = 0.258) (Table 4).

**Table 4:** Estimated duration and morbidity of hepaticojejunostomy

		Interrupted suture technique	Continuous suture technique	Interrupted + continuous suture technique		*p*-value
				Interrupted technique	Continuous technique	
Duration	Number	24	7	43	43	**0,002**
	- < 10 min	1 (4%)	5 (71%)	6 (14%)	11 (26%)	
	- 10-20 min	19 (79%)	2 (29%)	20 (47%)	25 (58%)	
	- 20-30 min	4 (17%)	0 (0%)	13 (30%)	6 (14%)	
	- > 30 min	0 (0%)	0 (0%)	4 (9%)	1 (2%)	
Morbidity	Number	23*	7	41*	41*	0,695
	- < 3%	7 (30%)	4 (57%)	9 (22%)	10 (24%)	
	- 3-5%	11 (48%)	1 (14%)	17 (41%)	16 (39%)	
	- 5-10%	5 (22%)	2 (29%)	9 (22%)	11 (27%)	
	- 10-15%	0 (0%)	0 (0%)	3 (7%)	2 (5%)	
	- 15-20%	0 (0%)	0 (0%)	3 (7%)	2 (5%)	
	- > 20%	0 (0%)	0 (0%)	0 (0%)	0 (0%)	
Leakage rate	Number	23*	7	42*	42*	0,258
	- < 3%	13 (57%)	5 (71%)	14 (33%)	15 (36%)	
	- 3-5%	10 (44%)	2 (29%)	19 (45%)	20 (48%)	
	- 5-10%	0 (0%)	0 (0%)	7 (17%)	6 (14%)	
	- 10-15%	0 (0%)	0 (0%)	2 (5%)	1 (2%)	

*Partially missing data due to incomplete answers

## Discussion

Hepaticojejunostomies are a common surgical procedure with a low complication rate, but relevant consequences in the event of complications. Various surgical techniques exist for the creation of a hepaticojejunostomy. So far, there is no randomized controlled comparison of techniques in the literature. Comparative data on the different techniques of hepaticojejunostomy are currently only available in the context of liver transplants (Table 5) [13, 14]. The results of these liver transplant studies suggest that an interruptedly sutured hepaticojejunostomy is associated with a higher leakage rate and the continuous sutured hepaticojejunostomy with a higher rate of stenosis [8]. Due to the small number of cases and the distinct indication, these results are likely to include relevant uncertainty and are therefore not transferable to common hepaticojejunostomies.

**Table 5:** Existing literature comparing hepaticojejunostomies in various techniques (Combi = combination of interrupted and continuous suture technique)

Author	Indication	Number	Follow-up	Technique	Number	Bile leak	Stenosis
Kasahara (2006) [13]	Liver transplantation	121	Median 60 months [7–80]	Interrupted	68	14,7%	7,4%
				Continuous	48	8,3%	10,4%
				Combi	5	20,0%	0,0%
Soejima (2006) [14]	Liver transplantation	76	3-year rate	Interrupted	53		31,8%
				Continuous	5		0,0%
				Combi	18		22,0%

This survey provides an overview of the surgical technique used for the creation of a hepaticojejunostomy in Germany. The results of the survey show a strong heterogeneity in the techniques used. The majority of respondents used both the interrupted suture as well as the continuous suture technique. This shows that even within most hospitals there is no standardization, but intraoperative reasons play the decisive role. The most common decision criterion among hospitals using both techniques is the bile duct diameter. This reflects the experience that in very small hepatic ducts the continuous suture technique can be very demanding. Moreover, the own particular surgical school will certainly play a crucial role.

The current survey suggests that the continuous suture technique is considered to be significantly faster, and both suture techniques are considered equivalent in terms of morbidity and, in particular, leakage rate. This raises the question why not all hepaticojejunostomies with adequate bile duct diameter are performed with the continuous suture technique. An adequate bile duct diameter should be present in most cases, since the bile duct is dammed up in the majority of cases due to the tumor. A randomized controlled comparison of the suturing techniques of interrupted suture technique and continuous suture technique is absolute necessary to answer this question.

An interesting aspect of the survey is the fact that university hospitals use significantly thinner sutures. In a review by Heidenhain in 2011, thin sutures are considered to be one of the decisive factors in the performance of a hepaticojejunostomy without complications [8]. However, the estimated complication rates of university hospitals and other hospitals do not differ in our survey. In addition, there are no comparative studies concerning the suture material.

This study has crucial limitations that need to be appropriately taken into consideration. Since data on the duration and complication rate of hepaticojejunostomies in this survey were given as estimates to facilitate participation in the survey, the validity of these data is limited. However, a very high response rate of 76% was achieved by a low threshold for participation in the survey. In addition, the estimated overall complication rate and the estimated insufficiency rate in the current survey are 3–5%. This value is comparable to the data published in the previous literature. This can underline a realistic assessment of the own complication rates and thus the value of the collected data. However, this could also be a sign that many respondents have answered the survey with known values from the literature and not their own realistic complication rate.

## Conclusion

In summary, heterogeneous techniques for hepaticojejunostomy are used in Germany. The most important decision criterion for the choice of technique is the bile duct diameter. The different techniques should be compared in a randomized controlled study.
